# Biotransformation of BDE-47 to Potentially Toxic Metabolites Is Predominantly Mediated by Human CYP2B6

**DOI:** 10.1289/ehp.1205446

**Published:** 2012-12-18

**Authors:** Maria Luisa Feo, Michael S. Gross, Barbara P. McGarrigle, Ethel Eljarrat, Damià Barceló, Diana S. Aga, James R. Olson

**Affiliations:** 1 Department of Chemistry, University at Buffalo, The State University of New York, Buffalo, NY, USA; 2 Department of Environmental Chemistry, Institute of Environmental Assessment and Water Research (IDAEA-CSIC), Barcelona, Spain; 3 Department of Pharmacology and Toxicology, University at Buffalo, The State University of New York, Buffalo, NY, USA; 4 Catalan Institute for Water Research (ICRA), Girona, Spain

**Keywords:** BDE-47, BDE metabolites, CYP2B6, human liver microsomes, hydroxylated metabolites, PBDE metabolism

## Abstract

Background: Previous studies have indicated that cytochrome P450s (CYPs) are involved in the metabolism of polybrominated diphenyl ether (PBDE) flame retardants in humans, resulting in the formation of hydroxylated PBDEs (OH-PBDEs) that are potentially more toxic than the parent PBDEs. However, the specific enzymes responsible for the formation of OH-PBDEs are unknown.

Objectives: The purposes of this study were to characterize the *in vitro* metabolism of 2,2´,4,4´-tetrabromodiphenyl ether (BDE-47) by human liver microsomes (HLM) and recombinant human CYPs, and to identify the CYP(s) that are active in the oxidative metabolism of BDE-47.

Methods: Recombinant human CYPs (CYP1A1, 1A2, 1B1, 2A6, 2B6, 2C8, 2C9, 2C19, 2D6, 2E1, and 3A4) were incubated with BDE-47 (20 µM), and the metabolites were measured and characterized using gas chromatography with tandem mass spectrometry (GC-MS/MS). For kinetic studies, CYP2B6 and pooled human liver microsomes (HLMs) were incubated with BDE-47 (0–60 µM).

Results: CYP2B6 was the predominant CYP capable of forming six OH-BDEs, including 3-OH-BDE-47, 5-OH-BDE-47, 6-OH-BDE-47, 4-OH-BDE-42, 4´-OH-BDE-49, and a metabolite tentatively identified as 2´-OH-BDE-66. On the basis of full-scan GC-MS analysis, we hypothesized the formation of two other metabolites: di-OH-tetra-BDE and di-OH-tetrabrominated dioxin. In kinetic studies of BDE-47 metabolism by CYP2B6 and pooled HLMs, we found *K*_m_ values ranging from 3.8 to 6.4 µM and 7.0 to 11.4 µM, respectively, indicating the high affinity toward the formation of OH-BDEs.

Conclusion: Our findings support a predominant role of CYP2B6 in the metabolism of BDE-47 to potentially toxic metabolites, including a hypothesized di-OH-tetrabrominated dioxin metabolite. These results will assist future epidemiological studies investigating the potential of PBDEs and their metabolites to produce neurobehavioral/neurodevelopmental disorders.

During the past decade both animal and human studies have supported an association between polybrominated diphenyl ether (PBDE) flame retardants and neurobehavioral/neurodevelopmental disorders, particularly following *in utero* and postnatal exposure (Costa and Giordano 2007; Darnerud et al. 2001; Eriksson et al. 2001; Herbstman et al. 2010; Roze et al. 2009; Suvorov et al. 2009). Although three mixtures of PBDEs have been produced globally (penta-BDE, octa-BDE, and deca-BDE), the lower brominated mixtures contain the more bioaccumulative and persistent congeners, with 2,2´,4,4´-tetrabromodiphenyl ether (BDE-47) representing the most abundant PBDE detected in human serum (Birnbaum and Staskal 2004). The use and production of penta-BDE and octa-BDE have been banned in Europe since 2004, and they were voluntarily phased out in the United States at the end of 2004. However, human and environmental levels of penta-BDEs are about 10-fold higher in North America than those in Europe or Asia, whereas levels of 2,2´,3,3´,4,4´,5,5´,6,6´-deca-BDE (BDE-209) are higher in Asia (Frederiksen et al. 2009; Law et al. 2008).

Although the ingestion of contaminated meat, fish, and dairy products is a known source of human exposure, the wide-spread use of PBDEs in household electrical appliances, plastics, televisions, drapes, and upholstered furniture has contributed to human exposure through inhalation and ingestion of household air and dust (Allen et al. 2007; Johnson et al. 2010). In contrast to other persistent organic pollutants, the highest body burdens of PBDEs are found in infants and toddlers, due primarily to exposure to maternal milk and house dust containing PBDEs (Costa et al. 2008; Fischer et al. 2006; Rose et al. 2010; Schecter et al. 2004). These findings are of concern since a study of New York City children ≤ 72 months of age found that lower scores on tests of cognitive, behavioral, and physical development were associated with higher levels of PBDEs in cord blood, which was used as a measure of perinatal exposure (Herbstman et al. 2010). In 6-year-old Dutch children, maternal PBDE levels in the 35th week of pregnancy were correlated with impairments in fine psychomotor abilities and attention, but also correlated with better coordination, better visual perception, and better behavior (Roze et al. 2009). In addition, Chevrier et al. (2010) reported an inverse association between PBDEs and serum thyroid-stimulating hormone (TSH) concentrations in pregnant women. These authors suggested that low TSH levels may result in maternal subclinical hyperthyroidism, which in turn has been associated with adverse pregnancy outcomes and potential effects on fetal and child development. Recently, hydroxylated metabolites of PBDEs (OH-PBDEs) have been found to accumulate in human serum at levels similar to, and in some cases greater than, that of the parent PBDEs (Athanasiadou et al. 2008; Qiu et al. 2009). The significance of this finding is heightened by mechanistic studies showing that monohydroxylated metabolites of BDE-47 are more potent than the parent BDE-47 in disrupting Ca^2+^ homeostasis, modulating γ-aminobutyric acid (GABA) and α4β2 nicotinic acetylcholine (nACh) receptor function, altering spontaneous activity and cell viability in cultured cortical neurons, and competing with thyroxine (T_4_) for binding to human transthyretin (TTR) (Dingemans et al. 2008, 2010a, 2010b, 2011; Hamers et al. 2008; Hendriks et al. 2010; Kim et al. 2011). Together, these and other studies suggest that bioactivation by oxidative metabolism adds considerably to the neurotoxic potential of PBDEs. Thus, there is a critical need to further our understanding of the factors affecting PBDE metabolism and accumulation of metabolites in humans.

Recently Stapleton et al. (2009) examined metabolism of 2,2´,4,4´,5-penta-BDE (BDE-99) and BDE-209 in fresh human hepatocytes. The authors reported the formation of monohydroxylated penta-BDE metabolites for BDE-99, whereas BDE-209 appeared not to be metabolized. Moreover, we previously demonstrated that human liver microsomes were capable of metabolizing BDE-47 and BDE-99 to OH-BDEs (Lupton et al. 2009, 2010). In studies of the oxidative metabolism of BDE-47 and BDE-99 in rat hepatic microsomes, OH-PBDE metabolites were identified for both BDE congeners (Erratico et al. 2011; Hamers et al. 2008).

The purpose of the present study was to qualitatively and quantitatively characterize the *in vitro* metabolism of BDE-47 using human liver microsomes and recombinant human cytochrome P450s (CYPs), and to identify the CYP(s) most active in the oxidative metabolism of this persistent BDE congener.

## Materials and Methods

*Standards and reagents*. We purchased human liver microsomes (HLMs) pooled from 50 donors from Xenotech (Lenexa, KS, USA). Baculovirus-insect cell microsomes (BD Supersomes) containing individually expressed human CYPs (CYP1A1, 1A2, 1B1, 2A6, 2B6, 2C8, 2C9, 2C19, 2D6, 2E1, 3A4) coexpressed with human CYP reductase and cytochrome b_5_, with characterized CYP protein levels and activities, were obtained from BD Biosciences (San Jose, CA, USA).

Commercially available standards of 2,2´,4-tribromodiphenyl ether (BDE-17), 2,4,4´-tribromodiphenyl ether (BDE-28), BDE-47, and BDE-99 used as internal standards, were purchased from Accustandard, Inc. (New Haven, CT, USA). A reference standard of ^13^C-labeled 6-hydroxy-2,2´,4,4´-tetra-BDE (^13^C-6-OH-BDE-47), used as surrogate, was obtained from Wellington Laboratories (Guelph, Ontario, Canada). Neat BDE-47 was purchased from Chem Service (West Chester, PA, USA).

The methoxylated (MeO) analog standards of 3-hydroxy-2,2´,4,4´-tetra-BDE (3-OH BDE-47), 5-hydroxy-2,2´,4,4´-tetra-BDE (5-OH-BDE-47), 6-hydroxy 2,2´,4,4´-tetra-BDE (6-OH-BDE-47), 4´-hydroxy-2,2´,4,5´-tetra-BDE (4´-OH-BDE-49) and 4-hydroxy-2,2´,3,4´-tetra-BDE (4-OH-BDE-42) were all gifts from M. Alaee (Environment Canada, Burlington, Ontario, Canada). Trimethylsilyl diazomethane, used for methylation of OH-PBDEs, was obtained from Sigma-Aldrich (St. Louis, MO, USA).

*CYP-specific metabolism of BDE-47.* Recombinant human CYPs, including CYP1A1, 1A2, 1B1, 2A6, 2B6, 2C8, 2C9, 2C19, 2D6, 2E1, and 3A4 (0.5 mg; 38.5 pmol CYP2B6 to 256 pmol CYP2C9), were individually incubated with 20 µM BDE-47 (dissolved in DMSO, 0.5% vol/vol) in 1 mL of buffer consisting of 50 mM HEPES, 6 mM magnesium chloride, 0.8 mg/mL bovine serum albumin (BSA) as a BDE carrier, and 1 mM NADPH at pH 7.4. This BDE concentration (20 µM) represented saturating conditions [see Supplemental Material, Figures S1 and S2 (http://dx.doi.org/10.1289/ehp.1205446)]. The assay was initiated with NADPH and incubated for 2 hr at 37°C to assess the potential of human CYPs to biotransform BDE-47. Samples were frozen and stored at –20°C until analysis. The following control samples were included in the study: BDE-47 incubations in the absence of microsomal protein (to determine any abiotic transformation of BDE-47), HLMs incubated in the absence of BDE-47 (to determine any background levels of OH-BDEs and MeO-BDEs), and recombinant CYPs incubated in the absence of BDE-47 (to monitor contamination). Control samples were prepared in duplicate.

*Kinetics for metabolism of BDE-47.* For kinetic studies, recombinant human CYP2B6 (40 pmol) and pooled HLMs (0.298 mg, 140 pmol total CYP) were incubated with BDE-47 (0.1, 0.25, 0.5, 1, 2.5, 5, 10, 20, 40, or 60 µM) for 60 min using the incubation conditions described above. Incubation time was previously determined to ensure linear formation of metabolites over time. Experiments were performed in triplicate. Using Origin 8 software (OriginLab Corporation, Northampton, MA, USA), we determined the kinetic values *V*_max_ and *K*_m,_ by nonlinear regression analysis of hyperbolic plots [i.e., velocity vs. substrate concentration (S)] obeying Michaelis–Menten kinetics.

*BDE-47 and metabolite extraction.* After incubation, each 1-mL sample was spiked with 0.2 µg ^13^C-BDE-47 and 0.2 µg ^13^C-6-OH-BDE-47, which served as surrogates. Samples were extracted with 3 mL 75/25 (vol/vol) solution of hexane/dichloromethane after adding 0.5 mL of 6 M HCl for protein denaturation. The mixtures were vortexed and centrifuged, and then the organic layer was removed from each sample. A second addition of 3 mL 50/50 (vol/vol) solution of hexane/dichloromethane was added, for extraction. The organic layer was removed and combined with the previous organic layer.

The extracts were evaporated to dryness under a stream of nitrogen and reconstituted in 0.4 mL 50/50 (vol/vol) hexane/dichloromethane. Then, 0.2 mL of each extract was collected and evaporated to dryness and reconstituted in 0.2 mL of toluene for analysis of BDE-47 by gas chromatography with tandem mass spectrometry (GC-MS/MS). We performed full-scan GC-MS analysis to check for the possible formation of debrominated metabolites.

The other 0.2-mL aliquot was used for the analysis of OH-BDE-47 metabolites by first derivatizing the sample with trimethylsilyl diazomethane. The reaction was performed for > 18 hr in a mixture containing 0.2 mL extract, 0.1 mL methanol, 0.05 mL hexane, and 0.25 mL trimethylsilyl diazomethane. After derivatization, samples were evaporated to dryness using a slow stream of nitrogen and then reconstituted in 3 mL hexane. A 3-mL aliquot of acetic acid (glacial acetic acid; Sigma Aldrich) was added to each sample to remove excess derivatizing agent. The mixture was vortexed and centrifuged to separate the organic layer. The organic layer was then transferred into a separate test tube, evaporated to dryness under a stream of nitrogen, and reconstituted with 0.1 mL toluene for analyses. Samples were analyzed under full-scan GC-MS to identify the metabolites, and then by selective reaction monitoring (SRM) GC-MS/MS to quantify the derivatized OH-PBDE metabolites.

*GC-MS and GC-MS/MS analysis.* The underivatized and derivatized extracts were analyzed using a Trace GC Ultra gas chromatograph coupled to a TSQ Quantum XLS triple quadrupole mass spectrometer both from Thermo Scientific (Waltham, MA, USA) and equipped with a programmable temperature vaporizing (PTV) injection system. All samples were monitored under electron impact ionization. Separation was performed on a Zebron Inferno™ ZB-5HT column (15 m × 0.25 mm i.d. × 0.10 µm film thickness; Phenomenex, Torrance, CA, USA). The PTV inlet had an initial temperature of 80°C, and was increased at a rate of 2.5°C/sec for 1 min. The carrier gas (helium) was set at 1.2 mL/min, and injection was in splitless mode.

For the analysis of PBDEs, we programmed the oven temperature to start at 100°C (held for 2 min), increase to 250°C at 25°C/min, and then increase to 300°C at 20°C/min. The oven temperature was held at 300°C for 5 min before it returned to the initial temperature. For the analysis of metabolites, the oven temperature started at 100°C (held for 2 min), increased to 250°C at 10°C/min, and then increased to 300°C at 15°C/min. The oven temperature was held at 300°C for 5 min, then returned to the initial temperature.

The GC-MS was initially operated under full-scan mode (scanning from *m/z* 100 to 650) to confirm the fragment ions (*m/z*) corresponding to BDE-47 and to determine molecular ions [M^•+^] of the PBDE metabolites formed. Once the *m/z* values were identified, GC-MS/MS analysis was performed under SRM mode (*m/z* 485→326 and 485→328 for BDE-47; *m/z* 516→356 and 516→358 for the OH-PBDE metabolites).

*Quality control.* For GC-MS/MS analysis, we determined extraction recoveries from samples spiked with BDE-47 (9.7 µg) containing heat-inactivated microsomes. Quantification was performed based on a standard calibration curve, using ^13^C-BDE-47 (0.2 µg) as a surrogate (added prior to extraction) to correct for recoveries. In addition, BDE-99 (0.2 µg) was added as internal standard (added after extraction and clean-up, just prior to GC-MS analysis) to check for instrument performance. Recoveries were between 100 and 110%. Samples were analyzed in duplicate.

To determine the overall derivatization and extraction recoveries for OH-PBDE metabolites, we spiked samples with a 0.2-µg aliquot of ^13^C-6-OH-BDE-47, which was then extracted and derivatized as described above. The recovery of ^13^C-6-OH-BDE-47 was quantified using an external standard calibration curve (6.25, 12.5, 25, 50, 100, 200 ng/mL) of 6-MeO-BDE-47. As described above, BDE-99 (5 ng) was used as the internal standard. The overall average spike recoveries were > 60%.

Other OH-PBDE metabolites in the samples were quantified based on external standard calibration curves (6.25, 12.5, 25, 50, 100, 200 ng/mL) using commercially available MeO-PBDE standards containing 3-MeO-BDE-47, 5-MeO-BDE-47, 6-MeO-BDE-47, 4´-MeO-BDE-49, and 4-MeO-BDE-42. Again, BDE-99 (5 ng) was used as the internal standard for these samples. All standards used for the calibration curve were prepared in toluene.

We determined method limits of detection (MLODs) and method limits of quantification (MLOQs) of the GC-MS/MS for the three OH-BDEs included in the kinetics study (3-OH-BDE, 5-OH-BDE, and 6-OH-BDE). MLOD and MLOQ are defined as the minimum amount of analyte that produces a peak with a signal-to-noise ratio equal to 3 and 10, respectively. Following the approach described by Harris (2011), the MLOD is about 2 pg, and the MLOQ is about 7 pg, for the three major OH-BDEs detected in this study.

## Results

*CYP-specific metabolism of BDE-47*. BDE-47 (20 µM) was incubated with 11 individual recombinant human CYPs (Table 1) for 120 min to assess the relative formation of monohydroxylated metabolites under the conditions described above. The relative amount of metabolite formed by each individual CYP tested is expressed in terms of the chromatographic peak area per nanomole of each respective CYP and expressed relative to the area formed by CYP2B6 (which was assigned as 100%). It is clear that CYP2B6 is the predominant CYP capable of metabolizing BDE-47 to the OH-BDEs identified in this study. Other CYPs that showed some activity toward PBDE oxidation, albeit at a much lesser extent, are CYP2C19 and CYP3A4.

**Table 1:** CYP-specific metabolism of BDE‑47 expressed as peak area/nmolCYP (% relative to CYP2B6).

Human CYP	3‑OH-BDE‑47	5‑OH-BDE‑47	6‑OH-BDE‑47	4’‑OH-BDE‑49	4‑OH-BDE‑42	2’‑OH-BDE‑66a
1A1 and 1A2	ND	ND	ND	ND	ND	ND
1B1	ND	ND	ND	ND	ND	ND
2A6	3.83 (100)	10.2 (100)	4.61 (100)	2.36 (100)	0.758 (100)	1.51 (100)
2B6	ND	ND	ND	ND	ND	ND
2C9	0.00805 (0.210)	ND	ND	0.00783 (0.332)	ND	ND
2C19	ND	ND	ND	ND	ND	ND
2E1	0.0507 (1.32)	0.0244 (0.239)	ND	ND	ND	ND
3A4	ND	ND	ND	ND	ND	ND
2D6	ND	ND	ND	ND	ND	ND
2C8	ND	ND	ND	ND	ND	ND
ND, not detected; peak areas < 1,000 were considered below the detection limit of GC‑MS/MS. Individual recombinant CYPs were incubated with 20 µM BDE‑47 for 120 min. aNo commercially available standard was available for 2’‑OH-BDE‑66; thus, it was tentatively identified and its identity cannot be confirmed.						

**Figure 1:**
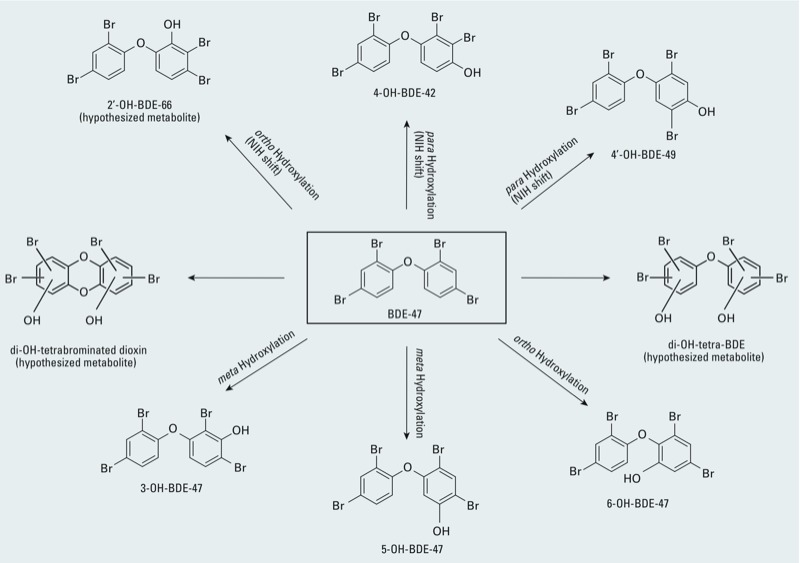
Metabolic products of BDE‑47 formed by CYP2B6. The structures of 2´‑MeO-BDE‑66, di‑OH-BDE, and di‑OH-tetrabrominated dioxin are hypothesized based on mass spectra data. NIH shift, intramolecular migration of a hydrogen atom primarily during hydroxylation.

**Figure 2:**
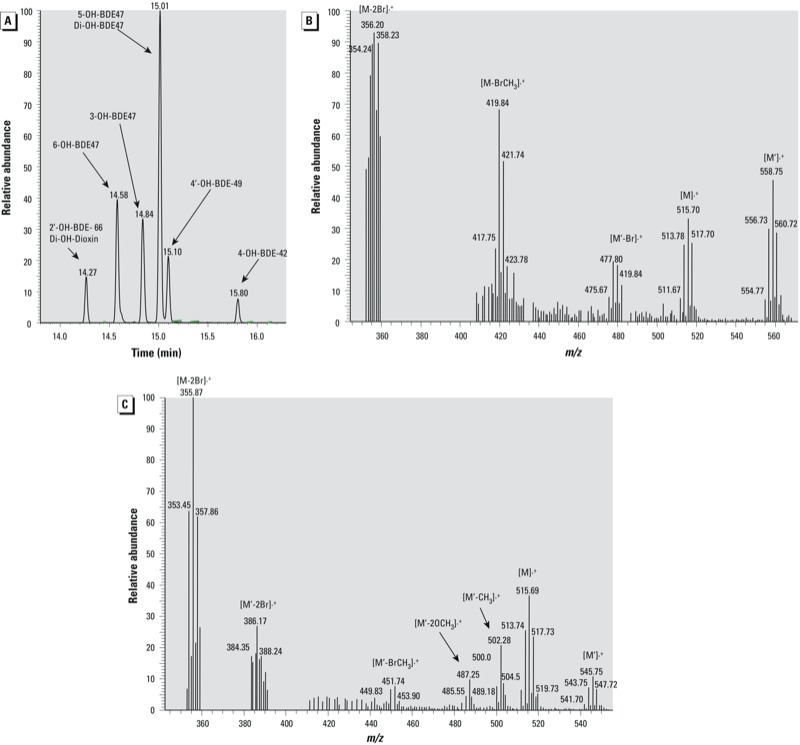
Results of GC-MS and GC-MS/MS analysis. (*A*) Chromatogram of the BDE‑47 metabolites monitored by GC-MS/MS in SRM mode. (*B,C*) Mass spectrum results from full-scan monitoring of (*B*) 2´-OH-BDE‑66 (M) and di‑OH-tetrabrominated dioxin (M´) and (*C*) 5- OH-BDE 47 (M) and di‑OH-BDE (M´). Because of the lack of commercially available standards for 2´-OH-BDE‑66, di‑OH-tetrabrominated dioxin, and di‑OH-BDE, the identity of these metabolites cannot be confirmed.

*Identification of metabolites.* Figure 1 shows the structures of the eight hydroxylated BDE-47 metabolites formed by recombinant human CYP2B6 and detected as methyl derivatives in the sample extracts. These metabolites were detected under full-scan GC/MS and were tentatively assigned their structures based on the MS isotopic signature of bromine. Later, the identities of five of these metabolites were confirmed by comparison of their retention times and full mass spectra with authentic methoxylated BDE standards (3-MeO-BDE-47, 5-MeO-BDE-47, 6-MeO-BDE-47, 4´-MeO-BDE-49, and 4-OH-BDE-42). Figure 2A shows a sample GC-MS/MS chromatogram in SRM mode of the derivatized OH-PBDEs formed during incubation of recombinant human CYP2B6 with BDE-47.

Analysis of metabolites formed by recombinant human CYP2B6-mediated oxidation of BDE-47 using full-scan GC-MS suggested the possible formation of two other metabolites: di-OH-tetra-BDE (retention time = 15.01; *m/z* = 545.75), which co-eluted with 5-OH-BDE-47 (Figure 2C); and di-OH-tetrabrominated dioxin (retention time = 14.27; *m/z* = 558.75), which co-eluted with a monohydroxylated metabolite tentatively identified as 2´-OH-BDE-66 (Figure 2B). The di-OH-tetra-BDE was also previously reported as one of the BDE-47 metabolites formed in HLM (Lupton et al. 2010). In contrast, di-OH-tetrabrominated dioxin has not been reported previously.

Because no standards were commercially available for 2´-OH-BDE-66, di-OH-tetrabrominated dioxin, or di-OH-tetra-BDE, their identities could not be confirmed. Structural analysis by nuclear magnetic resonance was not possible because of the limited amount of metabolites formed. However, the formation of 2´-OH-BDE-66 can be reasonably hypothesized based on the full mass spectra of the derivatized metabolite (Figure 2B) and on previous studies reporting the presence of this PBDE metabolite in human blood samples (Athanasiadou et al. 2008; Qiu et al. 2009) and in mice (Hamers et al. 2008; Qiu et al. 2007). Our hypothesis that 2´-OH-BDE-66 was formed—where the presence of OH is in the *ortho* position to the diphenyl ether bond—is supported by the full-scan mass spectra showing significant abundance of *m/z* 420 (loss of BrCH_3_). In a systematic study in which the fragmentation pattern of 26 methoxylated PBDEs was characterized by GC/MS, Athanasiadou et al. (2006) showed that all *ortho*-MeO-PBDEs undergo a loss of BrCH_3_ (M-94) in the electron impact ionization mode, which was absent or hardly observable for *meta*-MeO-PBDEs and *para*-MeO-PBDEs. Therefore, the presence of *m/z* 420 (M-94) in Figure 2B suggests that the derivatized OH-BDE formed an *ortho*-MeO-BDE corresponding to the proposed 2´-OH-BDE-66. Nevertheless, this proposed structure needs to be confirmed using authentic standards when available.

In control samples, no OH-PBDEs were detected in microsomal incubations in the absence of BDE-47. When underivatized samples prepared for the analysis and quantification of BDE-47 were analyzed for possible MeO-PBDE metabolites or contamination, no MeO-PBDEs were detected (data not shown). Possible metabolites formed by reductive debromination were observed in underivatized samples. However, reductive debromination was also observed in the control samples with BDE-47 incubated in the absence of microsomal proteins. All reductive debromination resulted in the formation of BDE-17 and BDE-28, which were identified based on their retention times and full mass spectra matching those of authentic reference compounds. Debromination present in the control samples was attributed to abiotic processes.

*Kinetic study*. We evaluated the formation of 3-OH-BDE-47, 5-OH-BDE-47, and 6-OH-BDE-47 [the three monohydroxylated BDE-47 metabolites, all of which were detectable at low BDE-47 concentrations (0.1 µM)] using recombinant human CYP2B6 and pooled HLMs incubated with BDE-47 (0.1–60 µM) for 60 min. Table 2 shows the kinetic parameters (*K*_m_ and *V*_max_) obtained from Michaelis–Menten plots based on the rate of OH-PBDE metabolite formation during incubation of BDE-47 with CYP2B6 and HLMs. After incubation of CYP2B6 and HLMs with BDE-47, *K*_m_ values for 3-OH-BDE-47, 5-OH-BDE-47, and 6-OH-BDE-47 were lower in CYP2B6 (6.4, 3.8, and 4.2 µM, respectively) compared with HLMs (11.4, 7.3, and 7.0 µM for 3-OH-BDE-47, 5-OH-BDE-47, and 6-OH-BDE-47, respectively) [see Supplemental Material, Figures S1 and S2 (http://dx.doi.org/10.1289/ehp.1205446)]. The lowest *K*_m_ values were observed for 5-OH-BDE-47 and 6-OH-BDE-47, the major metabolites produced by BDE-47 in both CYP2B6 (*V*_max_ = 948 pmol/min/nmol P450 and *V*_max_ = 202 pmol/min/nmol P450, respectively) and HLMs (*V*_max_ = 227 pmol/min/nmol P450 and *V*_max_ = 39.3 pmol/min/nmol P450, respectively) (Table 2).

**Table 2:** Comparison of kinetic parameters for the metabolism of BDE‑47 using recombinant human CYP2B6 and pooled HLMs.

Metabolite	CYP2B6		HLMs		
	Km (μM)	Vmax (pmol/min/nmol P450)	Km (μM)	Vmax (pmol/min/nmol P450)	Vmax (pmol/min/mg protein)
3-OH-BDE-47	6.4 ± 1.2	10.6 ± 0.6	11.4 ± 1.4	1.9 ± 0.1	0.91 ± 0.04
5-OH-BDE-47	3.8 ± 0.9	948 ± 56	7.3 ± 1.3	227 ± 12	107 ± 6
6-OH-BDE-47	4.2 ± 0.8	202 ± 10	7.0 ± 0.9	39.3 ± 1.5	18.5 ± 0.7
Values represent the mean ± SE of three experiments.					

## Discussion

Of the 11 recombinant CYPs that were screened, CYP2B6 was the predominant enzyme capable of transforming BDE-47 into 3-OH-BDE-47, 5-OH-BDE-47, 6-OH-BDE-47, 4-OH-BDE-42, 4´-OH-BDE-49, and a metabolite tentatively identified as 2´-OH-BDE-66 (Table 1). As illustrated in Figure 1, the formation of one *ortho*-hydroxylated metabolite (2´-OH-BDE-66) and two *para*-hydroxylated metabolites (4-OH-BDE-42 and 4´-OH´BDE-49) appear to result from an NIH shift (intramolecular migration of a hydrogen atom primarily during hydroxylation) of a bromine atom in concert with formation of an arene oxide intermediate (Boyd and Sharma 1996). Our results further suggest that CYP2B6 catalyzed the formation of two additional metabolites (Figure 1). One metabolite, dihydroxylated tetra-BDE (di-OH-tetra-BDE), has previously been reported (Lupton et al. 2010), but the present study is the first to suggest the formation of di-OH-tetrabrominated dioxin by CYPs. However, the identity of these metabolites cannot be confirmed because of the lack of commercially available standards. Moreover, we were unable to determine the specific location of bromination and hydroxylation in dihydroxylated tetra-BDE and di-OH-tetrabrominated dioxin because of the limited amount of metabolite formed. However, mass spectral data confirmed that both dihydroxylated metabolites contain four bromines, characteristic of the bromine isotopes.

With the exception of 2´-OH-BDE-66, all of the mono-OH-BDE metabolites detected in this study (Table 3) have been previously observed in human blood samples (Athanasiadou et al. 2008; Qiu et al. 2009), suggesting that the pathway for *in vitro* metabolism of BDE-47 by HLMs also reflects the *in vivo* biotransformation seen in humans.

**Table 3:** Metabolites of BDE‑47 previously found in humans.

Metabolite	Detected in human samples	Concentration (ng/g lipid weight)	Reference
3-OH-BDE‑47	Serum from children and young adults in Managua, Nicaragua	0.1–3	Athanasiadou et al. 2008
	Fetal blood from the United States	1.6	Qiu et al. 2009
	Maternal blood from the United States	0.1	Qiu et al. 2009
5-OH-BDE‑47	Fetal blood from the United States	28	Qiu et al. 2009
	Maternal blood from the United States	1.6	Qiu et al. 2009
6-OH-BDE‑47	Serum from children and young adults in Managua, Nicaragua	0.1–6	Athanasiadou et al. 2008
	Fetal blood from United States	9.9	Qiu et al. 2009
	Maternal blood from United States	0.3	Qiu et al. 2009
4´-OH-BDE‑49	Serum from children and young adults in Managua, Nicaragua	0.1–9	Athanasiadou et al. 2008
	Fetal blood from the United States	0.9	Qiu et al. 2009
	Maternal blood from the United States	0.3	Qiu et al. 2009
4-OH-BDE‑42	Serum from children and young adults in Managua, Nicaragua	0.15–5	Athanasiadou et al. 2008

It is important to note that OH-BDEs detected in human blood could also come from the diet and natural sources. In fact, OH-BDEs and MeO-BDEs have been identified as natural products produced by marine invertebrates and are present throughout the marine food web (Wiseman et al. 2011). OH-PBDEs also accumulate in the abiotic environment, including surface water, rainfall, snow, and wastewater and sediments, as possible products formed via the reaction of PBDEs with atmospheric hydroxyl radicals (Ueno et al. 2008). This means that OH-PBDEs in abiotic samples could be an important source for organisms, therefore influencing their concentrations in biota and humans through trophic transfer.

As presented in Table 1, CYP2C19 and CYP3A4 appear to be very minor contributors in the formation of OH-BDE metabolites from BDE-47. In contrast, CYP2B6 plays a major role in the formation of OH-BDE metabolites. Kinetic studies of BDE-47 metabolism by CYP2B6 and HLMs showed that the formation of 3-OH-BDE-47, 5-OH-BDE-47, and 6-OH-BDE-47 occurs at low BDE-47 concentrations, with apparent *K*_m_ values in the range of 3.8–6.4 µM and 7.0–11.4 µM, for CYP2B6 and HLMs, respectively. The relatively low *K*_m_ values suggest that CYP2B6 has high affinity for BDE-47, although low micromolar levels of BDE-47 are higher than those found in human tissues.

Recent mechanistic studies have shown that some OH-BDEs are more potent than parent compounds and may contribute substantially to neurodevelopmental disorders by direct neurotoxicity, or indirectly through thyroid disruption (Dingemans et al. 2008, 2010a, 2010b, 2011; Hamers et al. 2008; Hendriks et al. 2010; Kim et al. 2011). In contrast to BDE-47, which showed no activity, 6-OH-BDE-47 and 4´-OH-BDE-49 have been reported to be potent modulators of ryanodine receptors type 1 and 2, which regulate essential aspects of Ca^2+^ signaling (Kim et al. 2011; Pessah et al. 2010). Dingemans et al. (2008, 2010a, 2010b) reported that 6-OH-BDE-47 is more potent at mobilizing Ca^2+^ from endoplasmic and mitochondrial stores than is the parent compound. 6-OH-BDE-47 has also been reported to modulate human GABA_A_ and α4β2 nicotinic acetylcholine (nACh) receptors (Hendriks et al. 2010). These results are of added importance because CYP2B6 is present in most regions of the human brain (Miksys et al. 2003; Miksys and Tyndale 2004), and the local production of active metabolites may contribute directly to localized alterations in Ca^2+^ homeostasis, leading to altered brain development and neurodevelopmental disorders.

Six monohydroxylated metabolites of BDE-47 have also been shown to have thyroxine transport–disrupting activity (Marchesini et al. 2008). In fact, OH-PBDEs showed binding potency for thyroxine-binding globulin (TBG) and transthyretin (TTR), which are the two major thryroxine transport proteins in human plasma, each carrying 74% and 20% of total T_4_ (Cao et al. 2010; Marchesini et al. 2008). In particular, OH-PBDEs have been found to be moderate to strong binders to TTR and slight to moderate binders to TBG (Marchesini et al. 2008). Thus, OH-PBDEs are able to compete with the natural hormone T_4_ in binding either TTR or TBG (Marchesini et al. 2008).

Marchesini et al. (2008) reported that the 3-meta-OH group with two adjacent halogens present in 3-OH-BDE-47 provides the optimum structure for binding to TTR. These authors also reported affinity rankings for TTR (3-OH-BDE-47 > 5-OH-BDE-47 > 6-OH-BDE-47 > 4´-OH-BDE-49) and TBG (6-OH-BDE-47 > 3-OH-BDE-47 > 5-OH-BDE-47 > 4´-OH-BDE-49). Thus, the formation of mono-OH-BDEs in humans is an important issue because TTR is critical for maternal-to-fetal transport of thyroid hormones and for delivery of T_4_ across the brain barrier (Schreiber 2002), and because TBG has been linked to facilitation of the iodine supply to the fetus, which initially has no iodine reserve (Schussler 2000).

In addition to mediating BDE-47 metabolism, CYP2B6 exhibits up to 100-fold interindividual variability in hepatic protein expression as a result of regulatory phenomena and common genetic polymorphisms (Lang et al. 2001, 2004; Watanabe et al. 2010; Zanger et al. 2007). Thus, in addition to variable exposures to PBDEs, genetic variability in the CYP-specific metabolism of PBDEs may contribute to interindividual variability in the body burden of PBDEs and the formation of toxic metabolites.

## Conclusion

We identified CYP2B6 as the major enzyme involved in metabolism of BDE-47. Five of the mono-OH-BDE metabolites formed were identified based on comparison of their mass spectral fragmentation and GC-MS-MS retention times with commercially available standards. A sixth mono-OH-BDE metabolite was detected and tentatively identified as 2´-OH-BDE-66; however, its identity cannot be confirmed because of the lack of a commercially available standard. In addition, we tentatively identified a di-OH-tetra-BDE and a di-OH-tetrabrominated dioxin based on their mass spectral data; however, their identities can only be hypothesized and cannot be confirmed at this time because no standards are commercially available. These results will inform future mechanistic and epidemiological studies investigating the potential of PBDEs and their metabolites to produce neurobehavioral/neurodevelopmental disorders.

## Supplemental Material

(610 KB) PDF
