# Supplementary material for: Biotransformation of BDE-47 to Potentially Toxic Metabolites Is Predominantly Mediated by Human CYP2B6
Source: Environ Health Perspect. 2012 Dec 18;121(4):440–6. doi: 10.1289/ehp.1205446 (PMC3620761; doi:10.1289/ehp.1205446)
Supplement: (610 KB) PDF [file ehp.1205446.s001.pdf]

Supplemental Material

Biotransformation of BDE-47 to Potentially Toxic Metabolites Is Predominantly Mediated by Human CYP2B6

Maria Luisa Feo, Michael S. Gross, Barbara P. McGarrigle, Ethel Eljarrat, Damià Barceló, Diana S. Aga and James R. Olson.

List of Figures

Figure S1-A. Formation of 3OH-BDE-47 using recombinant CYP2B6……………………..2

Figure S1-B. Formation of 5-OH-BDE-47 using recombinant CYP2B6…………………….3

Figure S1-C. Formation of 6-OH-BDE-47 using recombinant CYP2B6…………………….4

Figure S2-A. Formation of 3OH-BDE-47 using human liver microsome…………………....5

Figure S2-B. Formation of 5-OH-BDE-47 using human liver microsome…………...............6

Figure S2-C. Formation of 6-OH-BDE-47 using human liver microsome…………………...7

A)

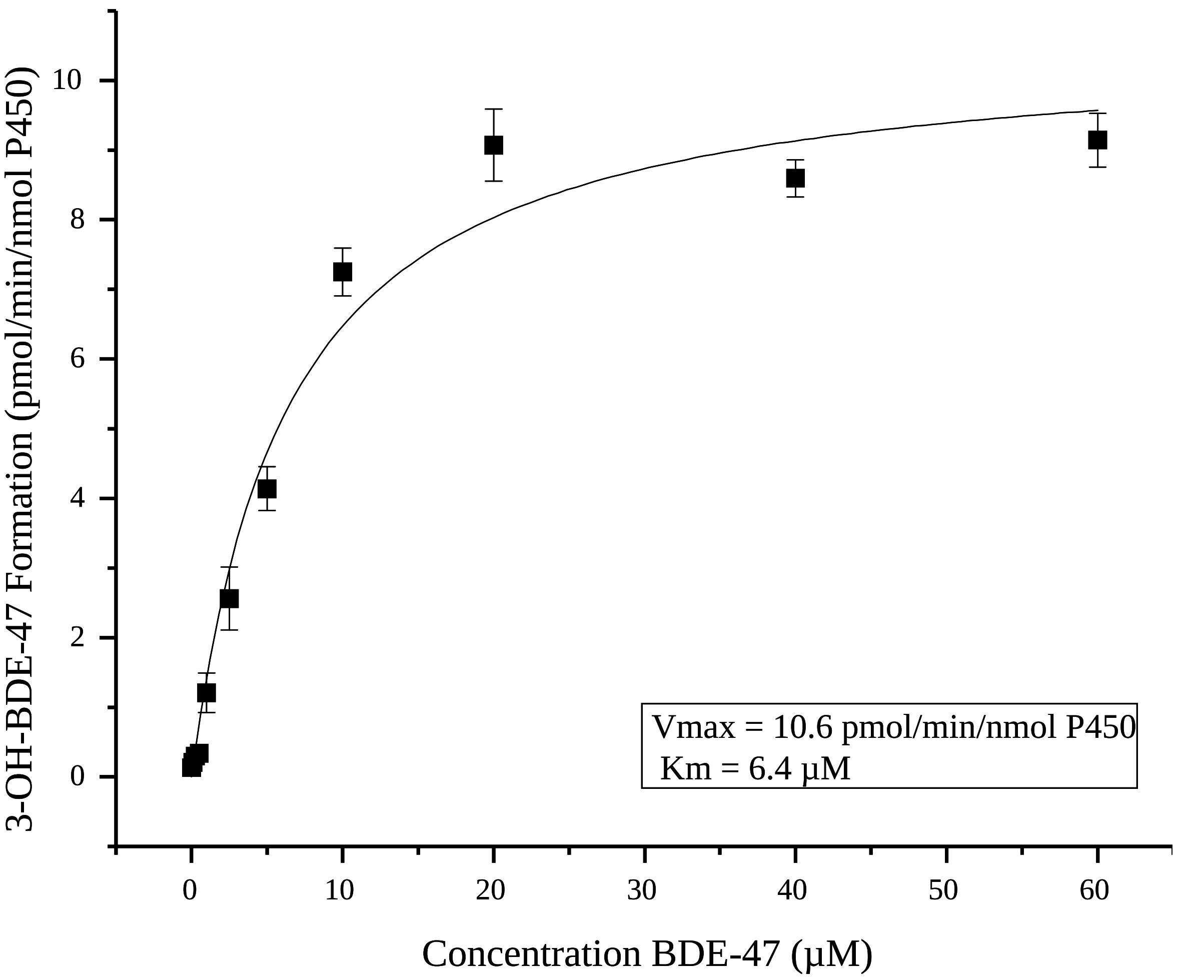
3-OH-BDE-47 Formation (pmol/min/nmol P450)
0
2
4
6
8
10
0
10
20
30
40
50
60
Concentration BDE-47 (µM)
Vmax = 10.6 pmol/min/nmol P450
Km = 6.4 µM

B)

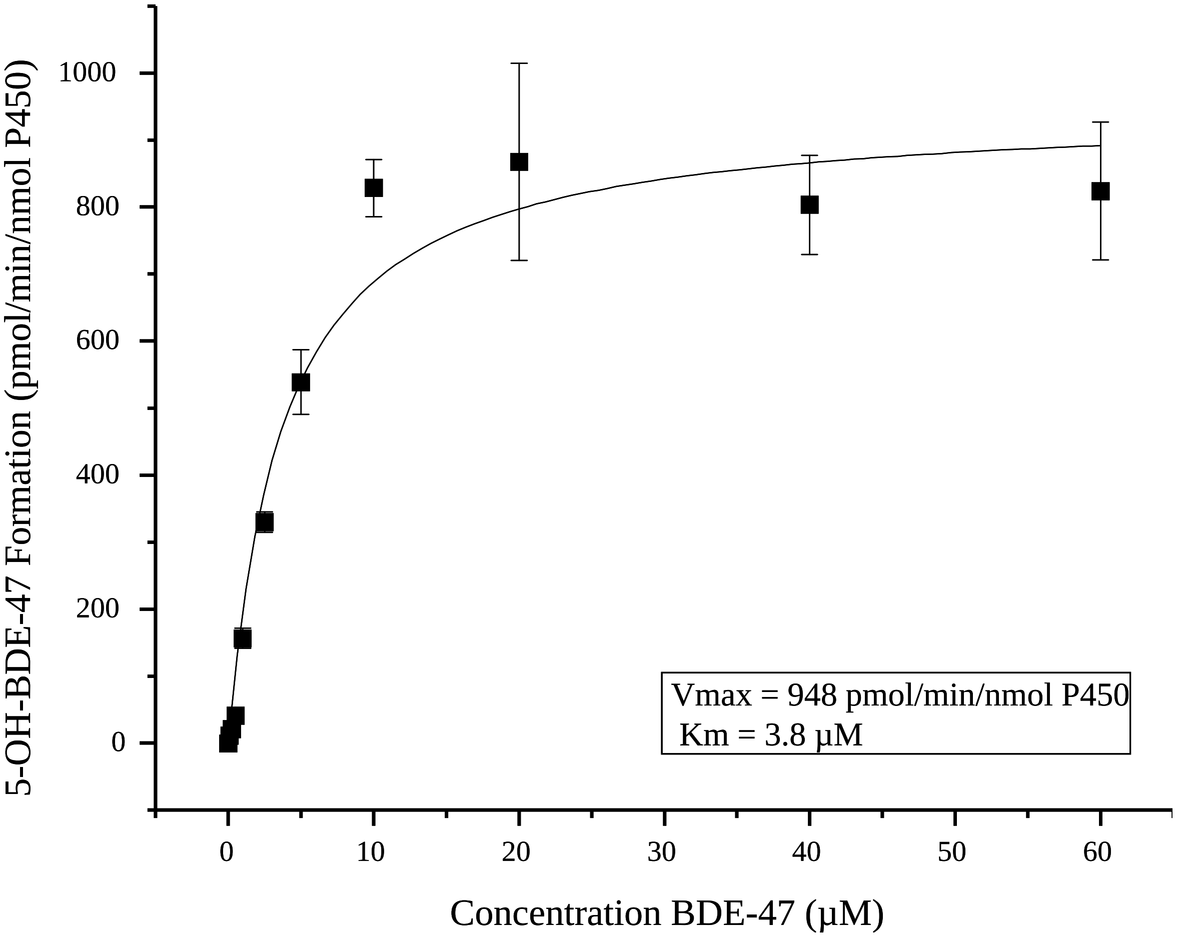
5-OH-BDE-47 Formation (pmol/min/nmol P450)
1000
800
600
400
200
0
0
10
20
30
40
50
60
Concentration BDE-47 (µM)
Vmax = 948 pmol/min/nmol P450
Km = 3.8 µM

C)

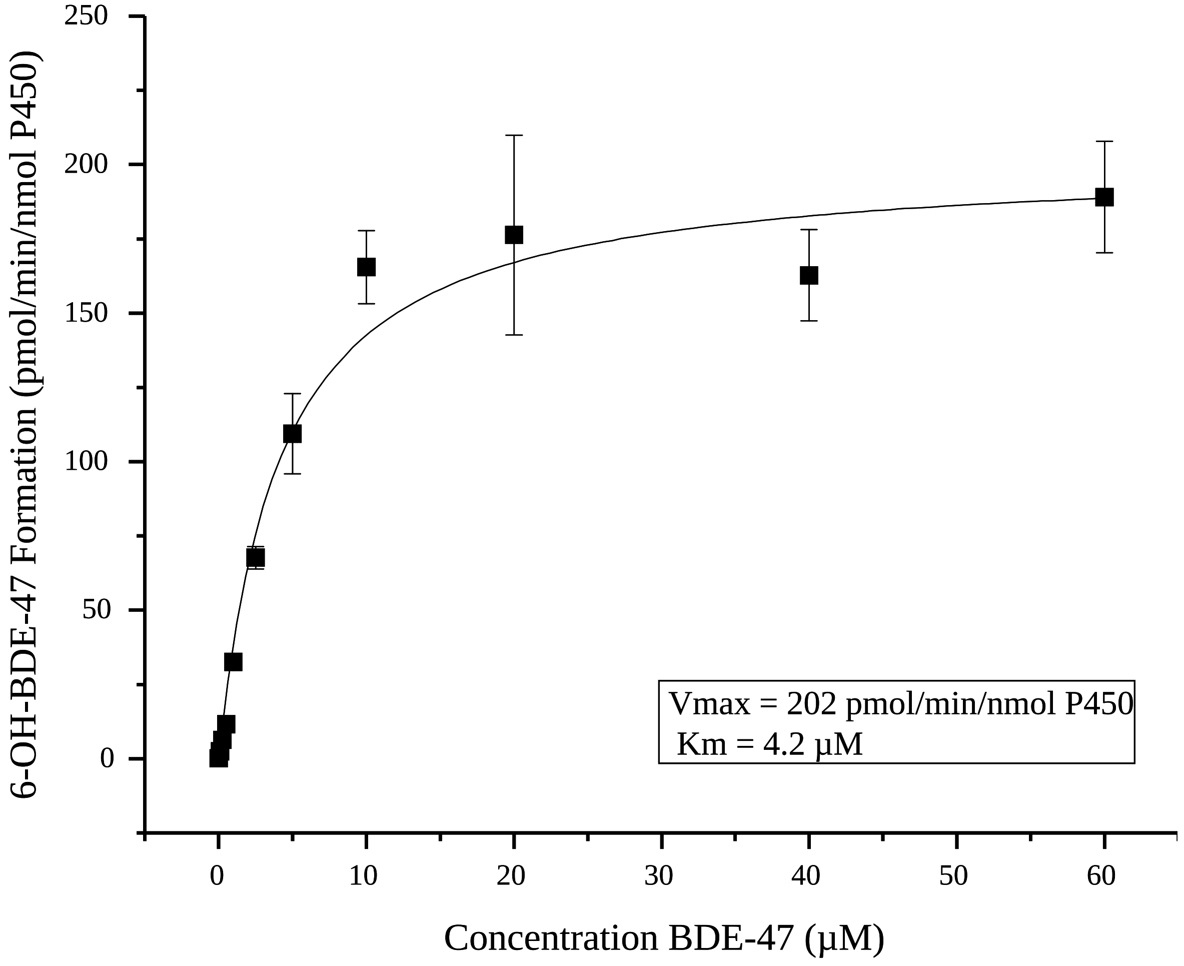

Supplemental Material, Figure S1. Formation of A) 3OH-BDE-47, B) 5-OH-BDE-47 and C) 6-OH-BDE-47 evaluated for over a range of substrate concentrations (0.1 to 60 µM) at an incubation time of 60 min using recombinant CYP2B6.

A)

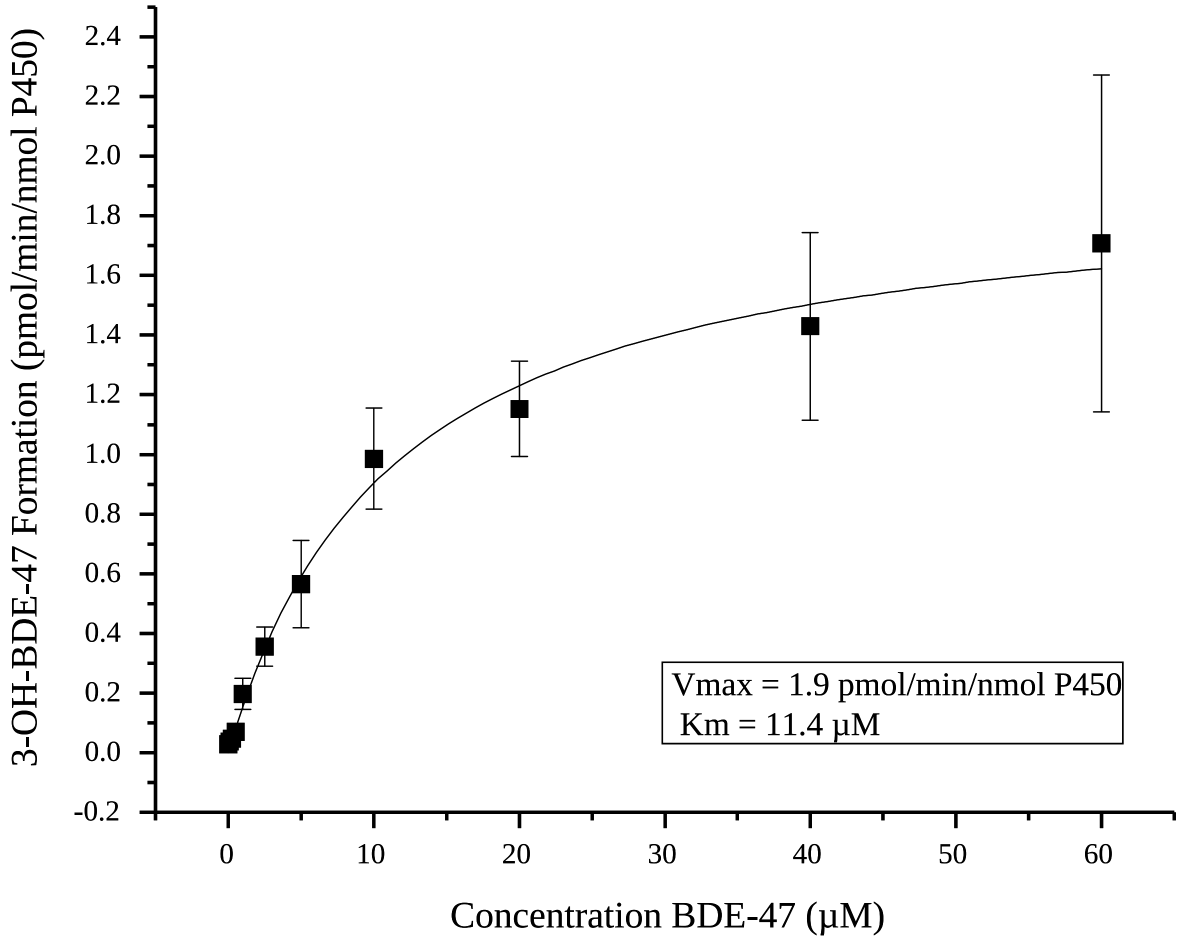
3-OH-BDE-47 Formation (pmol/min/nmol P450)
Concentration BDE-47 (µM)
Vmax = 1.9 pmol/min/nmol P450
Km = 11.4 µM
2.4
2.2
2.0
1.8
1.6
1.4
1.2
1.0
0.8
0.6
0.4
0.2
0.0
-0.2
0
10
20
30
40
50
60

B)

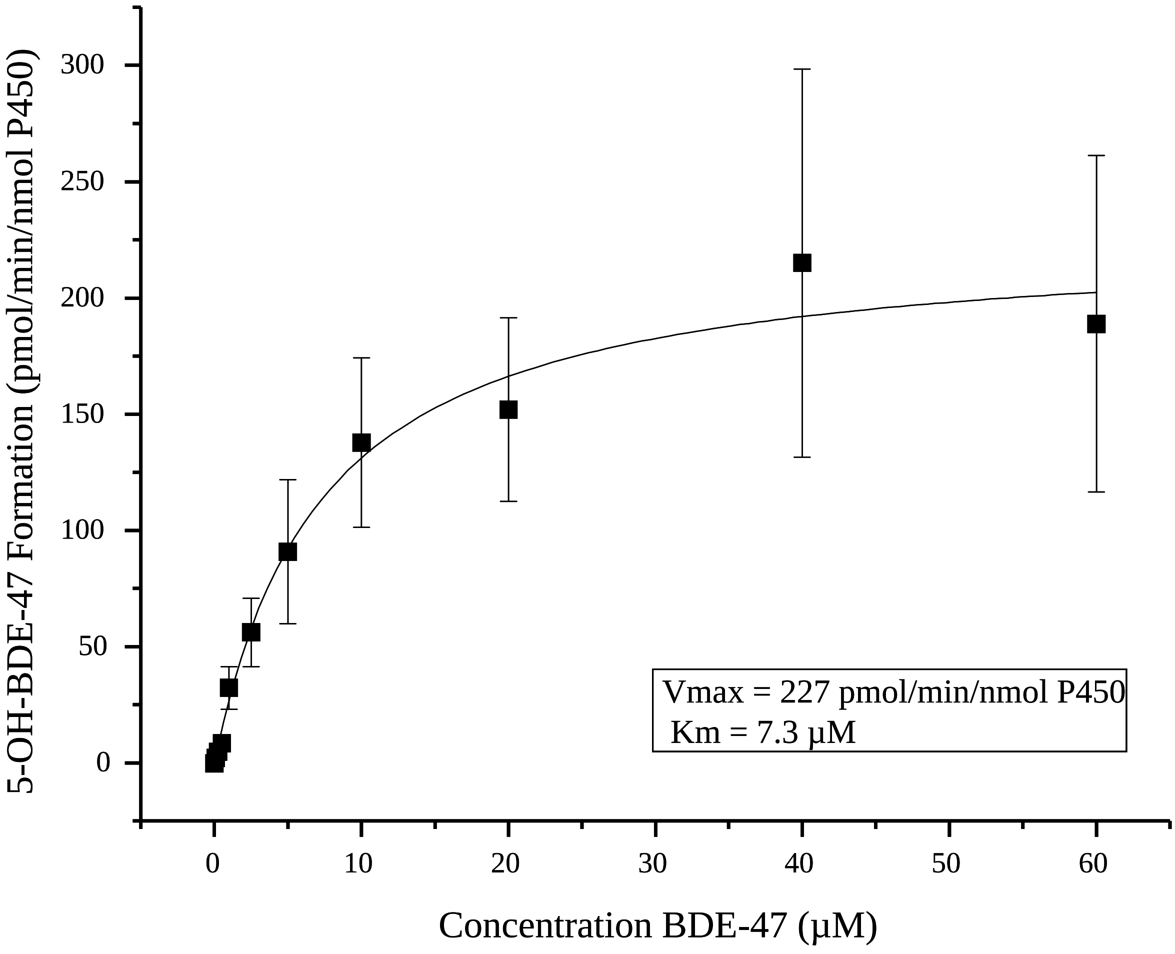

300
250
200
150
100
50
0
5-OH-BDE-47 Formation (pmol/min/nmol P450)
0
10
20
30
40
50
60
Concentration BDE-47 (µM)
Vmax = 227 pmol/min/nmol P450
Km = 7.3 µM

C)

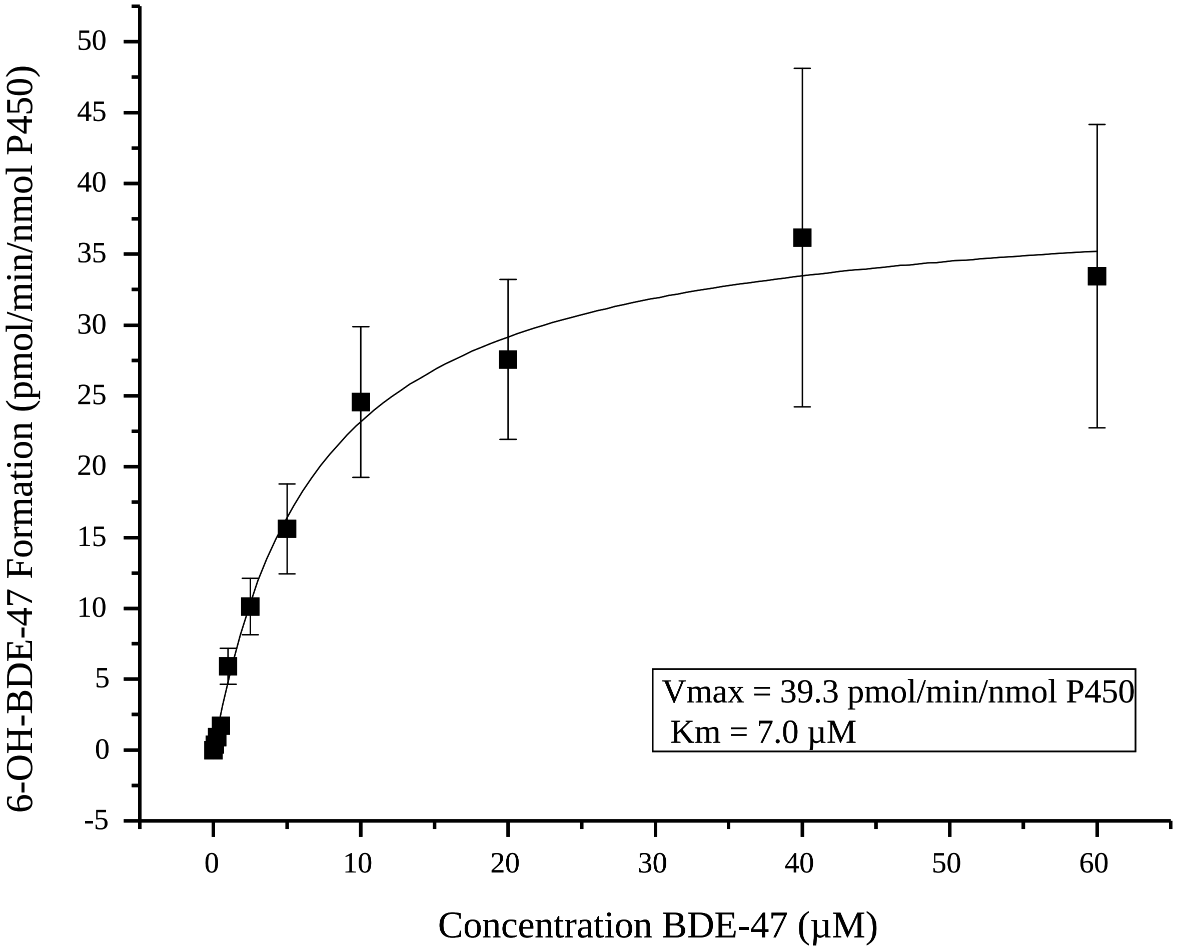

Supplemental Material, Figure S2. Formation of A) 3OH-BDE-47, B) 5-OH-BDE-47 and C) 6-OH-BDE-47 evaluated for over a range of substrate concentrations (0.1 to 60 µM) at an incubation time of 60 min using pooled human liver microsomes.
